# Effects of Two Weeks of Knee Compression After Total Knee Arthroplasty on Motor Function: A Controlled Before-and-After Study

**DOI:** 10.7759/cureus.95869

**Published:** 2025-10-31

**Authors:** Kunihiro Onishi, Shigeharu Tanaka, Kohei Matsumura, Yasushi Miura

**Affiliations:** 1 Department of Rehabilitation, Osaka Orthopedic Hospital, Osaka, JPN; 2 Department of Rehabilitation, Faculty of Health Science, Tokyo Kasei University, Sayama, JPN; 3 Department of Orthopedic Surgery, Tsukazaki Hospital, Himeji, JPN; 4 Department of Rehabilitation Science, Kobe University Graduate School of Health Sciences, Hyogo, JPN

**Keywords:** motor function recovery, postoperative swelling, post-surgical compression bandage, rehabilitation program, total knee arthroplasty

## Abstract

Objective

Swelling of the knee after total knee arthroplasty (TKA) impairs postoperative motor function. This study aimed to investigate whether knee swelling after TKA affects motor function and whether 2 weeks of compression intervention can improve postoperative motor recovery.

Methods

We screened 120 patients undergoing unilateral TKA. After exclusions and loss to follow-up, 88 patients were analyzed (compression group n=46; control group n=42). Outcomes (circumference, knee range of motion (ROM), pain, knee extensor strength, and walking speed) were assessed preoperatively and at 2 weeks after TKA. Statistical analyses were performed to compare the two groups.

Results

Swelling was significantly reduced in the compression group compared to the control group (36.4±2.6 cm in the compression group and 38.0±3.1 cm in the control group; p=0.014). Additionally, the ROM of knee extension (-1.1±2.1°in the compression group and -3.7±5.9°in the control group; p=0.015), pain at rest (6.1±15.1mm in the compression group and 15.9±19.1mm in the control group; p=0.001), muscle strength of knee extension (0.38±0.1Nm/kg in the compression group and 0.34±0.1Nm/kg in the control group; p=0.000), and gait speed (0.80±0.2m/s in the compression group and 0.75±0.2m/s in the control group; p=0.000) were significantly improved in the compression group. There was no significant difference in the ROM of knee flexion (113.6±13.0°in the compression group and 114.6±13.6° in the control group; p=0.708).

Discussion

To our knowledge, this is one of the few studies that have examined the effects of 2 weeks of knee compression on swelling and motor function after TKA. The compression group showed significantly higher improvement in motor function compared to the control group. Therefore, continuous compression for 2 weeks may be an effective method to reduce postoperative knee swelling and improve motor function after TKA.

Conclusion

It is suggested that compression of the knee for 2 weeks after TKA is useful in reducing swelling and improving motor function.

## Introduction

Knee osteoarthritis (KOA) is one of the major musculoskeletal diseases that often lead to physical limitation, pain, and decreased quality of life (QOL) [1]. Total knee arthroplasty (TKA) is an effective intervention, commonly performed in patients with end-stage KOA to relieve joint pain, improve mobility, and enhance QOL [2].

Swelling around the knee after TKA is a highly prevalent symptom [3,4]. Clinically, the swelling often interferes with activities of daily living (ADL) immediately after TKA, and patients often complain of heaviness and lack of strength in the legs. Previous interventional studies have attempted to improve the swelling [3,4,5,6,7]. There are studies that have examined the efficacy of various interventions for swelling [5,6], and most showed negative results [3,4,7]. However, it is presumed that the medical insurance system of each country has a significant impact on the length of hospitalization [8,9]. The average length of hospitalization for TKA in Western countries is approximately 3 days [8], whereas that in Japan is approximately 5 weeks [9]. The length of hospitalization inevitably limits the duration of knee compression intervention. Pinsornsak et al. [7] reported that compression had no effect on circumference and postoperative pain measured at 24 and 40 h after TKA. Yu et al. [10] reported that compression had no effect on circumference, postoperative pain, blood loss, or range of motion (ROM) measured 24 h after TKA. Additionally, Feng et al. [11] did not recommend compression bandage because it did not demonstrate improvements in blood loss, transfusion rate, ROM, or pain.

In previous studies [7, 10, 11], the duration of compression was short - 24 hours or less - and there were differences in the measurement methods of the knee circumference among the studies. Also, considerable variation may have occurred due to the material used for compression. Therefore, the effectiveness of compression longer than 24 h in a uniform method of compression and measurement, allowed only in long hospital stays, is unclear. The purpose of this study was to investigate whether postoperative knee swelling after TKA impairs motor function and to examine the effects of 2 weeks of compression intervention on swelling and postoperative motor recovery.

## Materials and methods

Patients and study design

**Figure 1:**
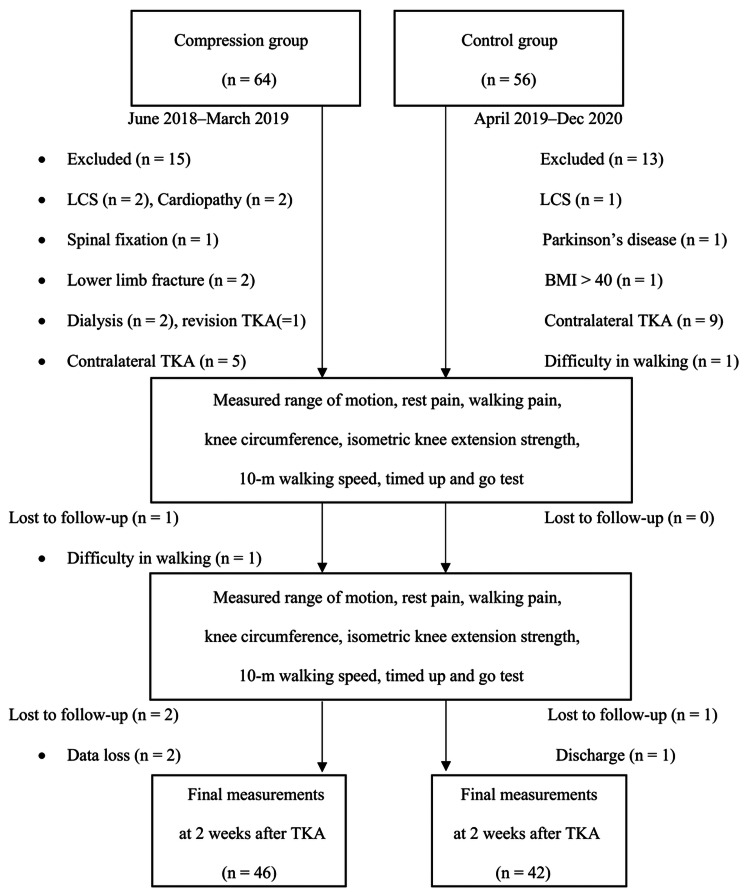
Design and flow of patients through the trial LCS, lumber canal stenosis; TKA, total knee arthroplasty; BMI, body mass index

A total of 120 patients who were diagnosed with medial knee osteoarthritis underwent unilateral TKA between June 2018 and December 2020. The inclusion criteria of this study were as follows: 1) patients who were hospitalized for TKA and participated in a rehabilitation program during hospitalization; and 2) those who could walk independently or with a cane for at least 20 m. The exclusion criteria were as follows: 1) lumbar spinal canal stenosis; 2) cardiopathy; 3) spinal fixation; 4) revision TKA; 5) lower limb fractures; 6) patients undergoing dialysis; 7) contralateral TKA; 8) Parkinson’s disease; 9) obesity; 10) difficulty in walking (Figure 1). The study design was a controlled before-and-after study. The compression group had 46 patients after the exclusion of 18, and the control group had 42 patients after the exclusion of 14 patients.

Surgical technique

The operation was performed under general anesthesia using an air tourniquet. Immediately before the procedure, 20 ml of 0.75% ropivacaine was injected into the subcutaneous tissue and intra-articular capsule. Using a medial parapatellar approach, the posterior cruciate ligament was resected, and a posterior-stabilized (PS) type Persona®︎ (Zimmer Biomet, Warsaw, USA) was deployed. The patella was replaced in all patients. After inserting a subcutaneous drain into the intra-articular capsule, 30 ml of 0.15% ropivacaine, 0.3 ml of 0.1% adrenaline solution, 3.8 mg of dexamethasone phosphate, and 0.5 mg of morphine hydrochloride were injected intra-articularly. All patients were prescribed edoxavantosylate for 2 weeks after TKA to prevent thrombosis. 

Compression of the knee after TKA

**Figure 2:**
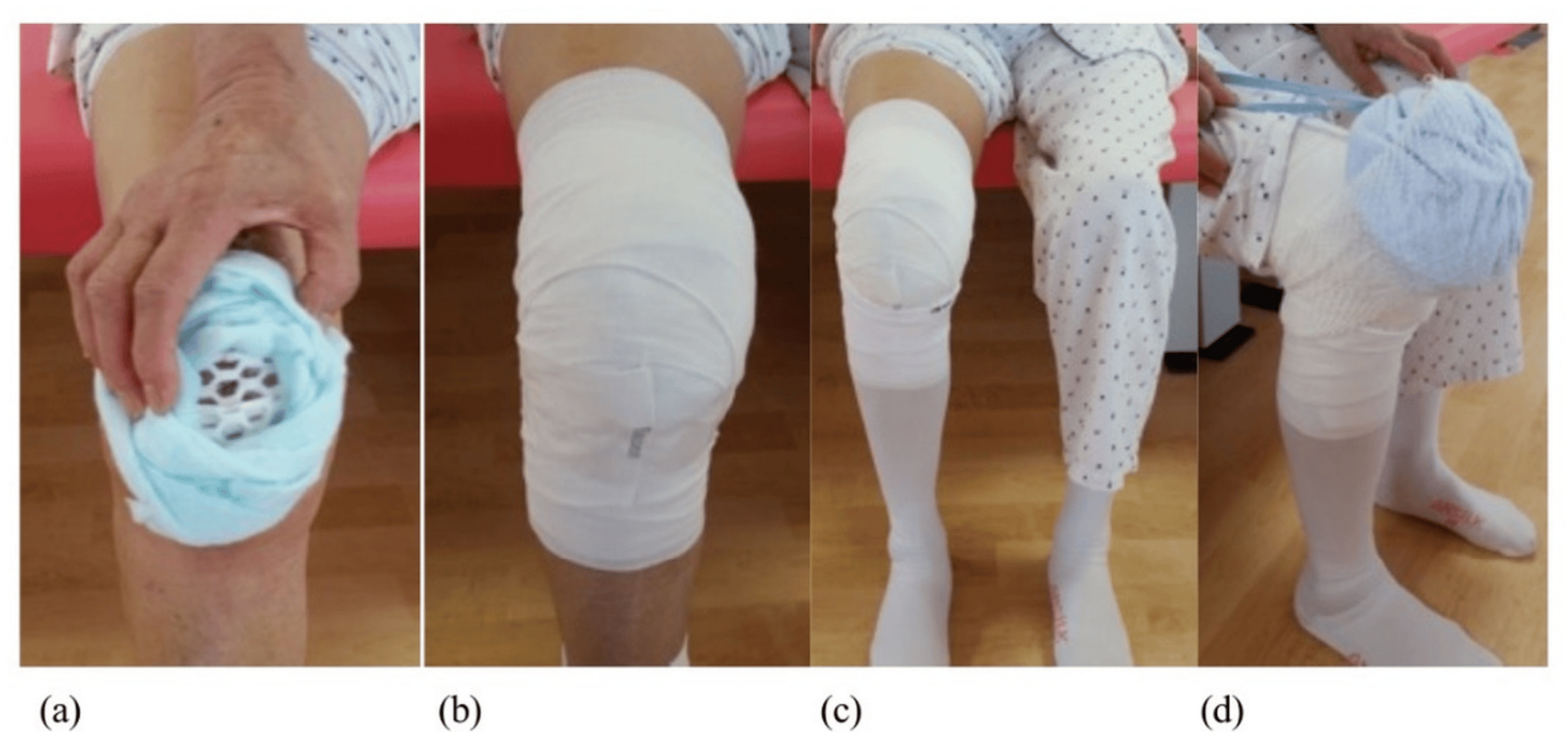
Methods of compression A cotton pad around the patella, held in place by an elastic bandage wrapped around the knee joint: (a) the cotton pad around the patella; (b) an elastic bandage wrapped across the knee joint; (c) an elastic stocking distal to the knee joint; (d) an ice pack on the knee joint.

In the compression group, a dressing with additional non-adherent absorbent padding (Opsite POST-OP Visible, Smith+Nephew, Tokyo, Japan) was applied on the wound after subcutaneous suturing. Thereafter, the patella was surrounded with a cotton pad (Oltex, ALCARE, Tokyo, Japan) and wrapped with an elastic cotton bandage (Elascot, ALCARE, Tokyo, Japan) from distal to proximal to equalize the pressure. This bandage was used for up to 2 weeks after TKA and then removed (Figure 2).

**Figure 3:**
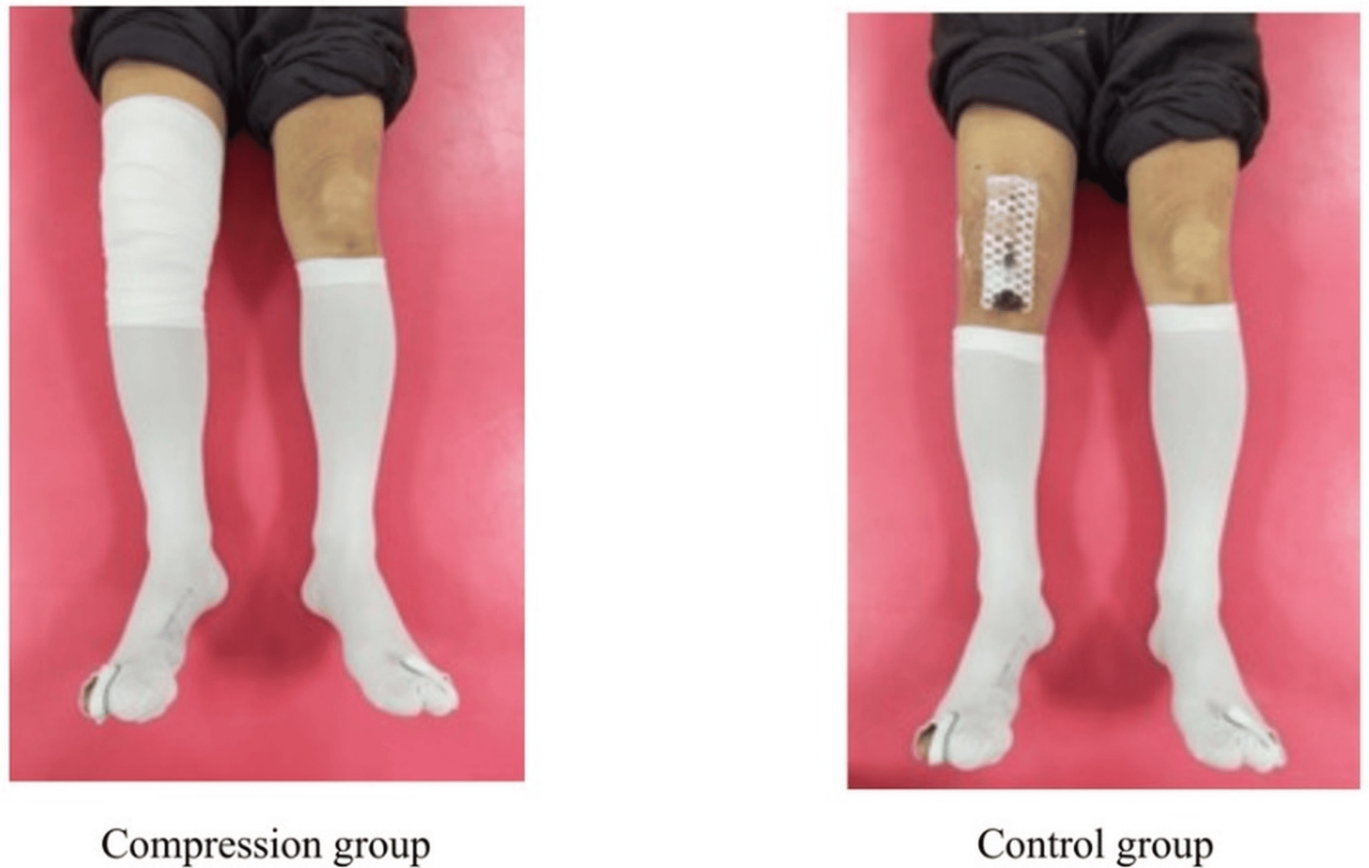
Postoperative management protocols in the control and compression groups The left panel shows an example of a patient in the compression group. The right panel shows an example of a patient in the control group. In the compression group, an additional cotton pad and elastic bandage were applied to the knee in combination with elastic stockings. In the control group, only elastic stockings were used. Both groups received elastic stockings postoperatively as mechanical prophylaxis for deep vein thrombosis.

In the control group, only a dressing with additional non-adherent absorbent padding (Opsite POST-OP Visible, Smith+Nephew, Tokyo, Japan) was applied on the wound after subcutaneous suturing (Figure 3).

In both groups, elastic stockings were worn by the patients postoperatively on both lower limbs immediately after TKA as mechanical prophylaxis for deep vein thrombosis. In addition, instructions for ankle pumping exercises were given before and after TKA, and walking was initiated from the first postoperative day.

Evaluation of swelling

Measurements were taken in the spine and knee extension position and at the circumference of the knee joint, 5 cm, and 10 cm above the base of the patella using a measuring tape. The reliability of the evaluation method has been examined in a previous study [12].

Motor function

In this study, the ROM of flexion-extension of the knee joint, rest pain, walking pain, isometric muscle strength of knee extension, and walking speed were measured preoperatively and 2 weeks after TKA. The ROM of active knee flexion and extension was measured in the supine position with full active knee ROM. The Knee ROM was measured using a plastic goniometer in 5° increments. The axis of the goniometer was placed over the lateral epicondyle of the femur, the proximal arm was aligned with the greater trochanter of the femur, and the distal arm was aligned with the lateral malleolus of the ankle. Knee pain was assessed using the 100 mm visual analog scale (VAS). Patients were questioned regarding the severity of knee pain at rest and during walking. Muscle strength of knee extension was measured using a hand-held dynamometer (HHD) (μTas F1; ANIMA, Chofu, Japan) with maximum isometric contraction on the involved side. Patients were seated with 90° flexion of the hip and knee joints and hands on their thighs, and the HHD was secured with a belt. The ankle cuff belt was located 3 cm proximal to the medial malleolus. The patients performed maximal isometric contractions of the knee extensors within 3 s. This test was performed three times, and the highest peak torque was selected for analysis. Isometric testing was performed on the involved side. The 10 m walking speed (10MWS) is a 10 m walking section with a 3 m interval between the acceleration and deceleration zones. The 10MWS was recorded, beginning when the patient’s lower limbs crossed the start line, and ending when the patient crossed the end line. It was measured at a preferred walking speed with the cane. The number of measurements was set to one. The value was indicated in m/min. 

Sample size

Sample size was calculated using the G*power version 3.1, a software program developed by Faul et al. [13]. The test family was the F test, and a statistical test was selected for repeated measurements within factors. The effect size f was set at 0.5, indicating a medium effect. The α error value was set at 0.05, and power was set at 0.80. As a result, a total of 68 patients (34 patients per group) were required for this study.

Statistical analysis

Baseline demographic and clinical characteristics (age, sex, body mass index (BMI), affected side, Kellgren-Lawrence grade, preoperative and postoperative femorotibial angle (FTA), use of edoxaban, and number of hospital days) were compared between the compression and control groups. Continuous variables with a normal distribution (age, BMI, FTA, and hospital days) were analyzed using the unpaired Student’s t-test, whereas non-normally distributed variables (knee circumference, range of motion, pain, knee extension strength, and 10MWS) were analyzed using the Mann-Whitney U test. Categorical variables (sex, affected side, Kellgren-Lawrence grade, and use of edoxaban) were analyzed using the chi-square test to examine differences in distribution between the groups. Additionally, the effect size was calculated for each factor. Hedges’ G effect size was considered small (0.2), medium (0.5), or large (≥0.8) [14]. Statistical significance was set at α = 0.05. All statistical analyses were performed using SPSS software version 22 (IBM SPSS, Tokyo, Japan). A p-value < 0.05 was considered statistically significant. 

Ethics statement

This study was conducted after receiving approval from the Ethics Review Committee of Tsukazaki Hospital (approval number: 191019, approval date: 18 June 2018), and all participants gave informed written consent.

## Results

Table 1 shows the basic information of the two groups. Postoperative FTA was significantly higher in the control group (174.5 ± 2.5° in the compression group and 176.2 ± 2.1° in the control group; p<0.01). There were no other significant differences.

**Table 1:** Characteristics of patients in the compression and control groups Data are presented as average ± standard deviation of the mean. Continuous variables were compared using the unpaired Student’s t-test or Mann-Whitney test for non-normally distributed data. Categorical variables are compared using the χ2 test. The p-value and effect size are shown. BMI, body mass index; **p<0.01

	Compression group	Control group	P value	Effect size
Age (years)	75.0 ± 6.2	73.8 ± 9.9	0.927	0.137
Gender (females/males)	7/39	10/32	0.308	0.108
Side (right/left)	27/19	25/17	0.937	0.008
BMI (kg/m^2^)	25.7 ± 4.0	26.0 ± 4.5	0.910	0.067
Kellgren–Lawrence grade	II:1 III:32 IV:13	II:1 III:28 IV:13	0.997	0.008
Preoperative femorotibial angle (°)	181.3 ± 7.1	182.0 ± 6.5	0.541	0.108
Postoperative femorotibial angle (°)	174.5 ± 2.5	176.2 ± 2.1	0.000**	0.733
Edoxaban tosilate hydrate	46	42	1.000	0.000
Number of hospital days	19.3 ± 5.1	23.0 ± 6.4	0.009**	0.623

Table 2 shows the baseline characteristics of knee circumference and motor function. The circumference of the knee joint at any of the measurement points and the motor function were not significantly different between the two groups.

**Table 2:** Preoperative comparison between the compression and control groups Data are presented as average ± standard deviation of the mean. Continuous variables were compared using the unpaired Student’s t-test or Mann-Whitney test for non-normally distributed data. The p-value and effect size are shown. ROM, range of motion; 10MWS, 10-m walking speed

	Compression group	Control group	P value	Effect size
Circumference of the knee (cm)	34.6 ± 2.5	35.8 ± 3.2	0.148	0.046
5 cm above the patella (cm)	40.5 ± 4.2	41 ± 4.8	0.548	0.128
10 cm above the patella (cm)	43.9 ± 4.4	44.2 ± 5.2	0.800	0.054
Uninvolved knee flexion ROM (°)	129.8 ± 10.9	128.8 ± 12.1	0.796	0.083
Involved knee flexion ROM (°)	120.2 ± 13.9	121.1 ± 16.4	0.795	0.056
Uninvolved knee extension ROM (°)	−3.5 ± 5.5	−3.2 ± 5.5	0.845	0.047
Involved knee extension ROM (°)	−6.3 ± 7.5	−6.7 ± 8.2	0.881	0.046
Rest pain (mm)	7.8 ± 16.9	10.5 ± 17.7	0.227	0.152
Walking pain (mm)	53.1 ± 22.5	44.4 ± 23.9	0.088	0.368
Involved knee extension strength (Nm/kg)	0.80 ± 0.3	0.78 ± 0.3	0.748	0.073
10 MWS (m/s)	0.77 ± 0.3	0.61 ± 0.4	0.081	0.475

Table 3 shows the comparison of circumference and motor function between the groups at 2 weeks after TKA. The circumference of the knee joint was significantly decreased in the compression group (36.4 ± 2.6 cm in the compression group and 38.0±3.1 cm in the control group; p<0.05), indicating a medium effect size. The other measurements of circumference, at 5 cm (42.7 ± 4.1 cm in the compression group and 43.2 ± 5.1 cm in the control group; p=0.642) and 10 cm (45.5 ± 4.1 cm in the compression group and 45.8 ± 5.3 cm in the control group; p=0.797) above the patella, and the ROM of knee flexion (113.6 ± 13.0° in the compression group and 114.6±13.6° in the control group; p=0.708) did not significantly differ. The ROM of knee extension, as well as rest pain, was significantly improved in the compression group (-1.1 ± 2.1° in the compression group and -3.7 ± 5.9° in the control group;　p<0.01), showing a medium effect size. There were no significant differences in walking pain (19.7 ± 19.9 mm in the compression group and 26.0 ± 22.5 mm in the control group; p=0.241). The muscle strength of knee extension and 10MWS improved significantly in the compression group (0.80 ± 0.2 m/s in the compression group and 0.75 ± 0.2 m/s in the control group; p<0.01), showing a small and large effect size, respectively.

**Table 3:** Comparison between the compression and control groups at 2 weeks after TKA Data are presented as average ± standard deviation of the mean. Continuous variables were compared using the unpaired Student’s t-test or Mann-Whitney test for non-normally distributed data. The p-value and effect size are shown. TKA, total knee arthroplasty; ROM, range of motion; 10MWS, 10-m walking speed; **p<0.01;  *p<0.05

	Compression group	Control group	P value	Effect size
Circumference on the knee (cm)	36.4 ± 2.6	38.0 ± 3.1	0.014*	0.532
5 cm above the patella (cm)	42.7 ± 4.1	43.2 ± 5.1	0.642	0.099
10 cm above the patella (cm)	45.5 ± 4.1	45.8 ± 5.3	0.797	0.055
Involved knee flexion ROM (°)	113.6 ± 13.0	114.6 ± 13.6	0.708	0.078
Involved knee extension ROM (°)	−1.1 ± 2.1	−3.7 ± 5.9	0.015*	0.584
Rest pain (mm)	6.1 ± 15.1	15.9 ± 19.1	0.001**	0.564
Walking pain (mm)	19.7 ± 19.9	26.0 ± 22.5	0.241	0.296
Involved knee extension strength (Nm/kg)	0.38 ± 0.1	0.34 ± 0.1	0.000**	0.239
10 MWS (m/s)	0.80 ± 0.2	0.75 ± 0.2	0.000**	0.728

## Discussion

Previous studies have primarily evaluated short-duration compression (≤24 h) with heterogeneous measurement protocols and materials, leaving the effectiveness of longer, standardized compression uncertain. In this context, we examined a 2-week postoperative compression protocol after TKA and observed that the compression group had greater improvements in motor function and reduced knee swelling than the control group. These findings suggest that continuous compression over 2 weeks, when applied with a uniform method, may help mitigate postoperative swelling and may be associated with better motor function after TKA.

Many strategies have been used to treat swelling after TKA. Previous studies on compression after TKA [3,4,7,13] have concluded that compression has no effect on the outcome and is not necessary. Randomized controlled trials and meta-analyses on swelling reported no effects of compression on reducing swelling or pain, or increasing the ROM [7,11]. The difference between this study and the previous studies is the duration of compression: 2 weeks in the present study compared to a significantly shorter duration of 24-48 h in the previous studies [7,10,11]. 

This study showed significant improvement in the ROM of knee extension in the compression group compared to the control group at 2 weeks after TKA. Mizner et al. [15] reported a knee extension ROM of -2° 1 month after TKA. The use of cotton pads around the patella to equalize the pressure under compression might minimize postoperative swelling and result in early improvement, especially in the ROM of knee extension. As a mechanism for reducing this swelling, Charalambides et al. [5] and Ramelet [16] reported that the tamponade effect of the knee reduces intra-articular bleeding and increases intra-articular pressure in the knee joint, thereby reducing soft tissue edema.

Pain [17], surgical trauma [18], postoperative knee swelling [19], and arthrogenic muscle inhibition (AMI) [20] have been reported as causes of muscle weakness after TKA. It has also been reported that early postoperative muscle weakness is not due to pain, but mainly due to inadequate activation of voluntary muscles [21]. Moreover, at 1 month after TKA, muscle strength in the quadriceps muscle on the operative side decreases to half of the preoperative level [21,22]. In our study, the muscle strength was 40% of the preoperative level at 2 weeks after TKA, indicating earlier improvement than previous studies [21,22]. In particular, a previous study [22] considered AMI to be one of the causes of muscle weakness in the early postoperative period. Hopkins et al. [23] and McNair et al. [24] reported that knee effusion and swelling stimulate mechanoreceptors in the knee joint, which in turn inhibit lower motor neurons and the quadriceps muscles. Therefore, this study suggests that compression early in the postoperative period could control intra-articular hemorrhage, reduce swelling, and prevent muscle weakness due to AMI.

There are many possible reasons for improvement in walking speed, including various factors such as pain, muscle strength, and ROM. Stevens et al. [22] and Mizner et al. [21] have reported that muscle contraction activation failure is rarely caused by knee pain during muscle contraction after TKA. Therefore, pain-induced decreased muscle exertion may not play a significant role in walking. In this study, walking pain was not significantly different between the two groups, being approximately 20% of the intensity; therefore, it is unlikely that pain affected walking speed. Pua et al. [25] reported that quadriceps muscle strength up to 16 weeks after TKA is the strongest predictor of gait speed. In our study, knee extension muscle strength was significantly improved in the compression group compared to the control group. Additionally, owing to the correlation between quadriceps muscle strength and functional performance after TKA [15,21], functional performance is expected to improve in accordance with quadriceps muscle strength. In contrast, knee joint ROM and gait in patients who have undergone TKA are related to knee flexion angle [26,27,28]. The maximum knee flexion angle required for walking is 67° [29]; however, in this study, both groups gained more than 110°. The present study suggests that compression is more effective in improving knee extension ROM than flexion. Tarnita et al. [30] reported that the knee flexion angle increased more significantly when the walking speed was increased. However, the relation between a greater knee ROM and faster walking speed remains unclear; therefore, further research is needed in this regard.

This study evaluated short-term outcomes at 2 weeks postoperatively; therefore, the observed differences may not necessarily reflect long-term clinical benefits. The primary aim of this study was to examine whether continuous knee compression during the early postoperative phase could influence recovery, a period characterized by pronounced swelling and reduced motor function after TKA. Early improvements in swelling reduction, knee extension range of motion, and quadriceps strength may contribute to smoother progress in subsequent rehabilitation and functional recovery. However, the clinical importance of these early improvements remains uncertain without long-term follow-up. Therefore, future studies are warranted to investigate whether the effects of compression persist over extended postoperative periods and lead to measurable long-term functional benefits.

This study has some limitations. First, this was a single-center study, which limits the generalizability of the findings. Second, the study employed a controlled before-and-after design rather than a randomized controlled trial, making it difficult to eliminate potential confounding factors. Third, although the sample size was calculated using G*Power, a relatively high number of exclusions may have reduced the actual statistical power. Fourth, although the compression method was standardized, the pressure applied was not quantitatively measured, raising concerns about reproducibility. Fifth, the follow-up period was limited to 2 weeks, and long-term outcomes such as swelling, muscle strength, or ADL recovery at 6 months or 1 year were not assessed. Sixth, the allocation of patients into groups was based on admission period, meaning changes in hospital environment or rehabilitation protocols over time could have influenced the results.

## Conclusions

This study examined whether 2 weeks of knee compression after TKA affects postoperative swelling and motor function. The results suggest that continuous knee compression for 2 weeks may help reduce swelling and improve early postoperative motor function. However, as this was a short-term and retrospective study, the clinical implications of these findings remain uncertain. Further prospective and long-term studies are warranted to determine whether early improvements in swelling and motor recovery lead to meaningful and sustained functional benefits.
